# The Influence of Socioeconomic Status on Adolescent Aggressive Behavior: The Mediating Roles of Normative Beliefs About Aggression and Aggressive Affect

**DOI:** 10.3390/bs16030446

**Published:** 2026-03-18

**Authors:** Yuhan Yang, Xu Wang, Youling Bai, Yanling Liu

**Affiliations:** 1 Faculty of Psychology, Southwest University, No. 2, Tiansheng Road, Beibei District, Chongqing 400715, China; yyh1128@email.swu.edu.cn (Y.Y.); wangxu010550@email.swu.edu.cn (X.W.); byling819@email.swu.edu.cn (Y.B.); 2 Mental Health Education Research Center, Southwest University, Chongqing 400715, China

**Keywords:** socioeconomic status, aggressive behavior, physical aggression, verbal aggression, relational aggression, gender differences

## Abstract

Adolescence is a period in which the frequency of aggressive behavior increases. However, the relationship between socioeconomic status (SES) and different types of aggression remains unclear. This study explored this relationship among middle school students. It also examined the mediating mechanisms of aggressive cognition and affect, while investigating the gender differences in the relationships among the study variables. A total of 1891 middle school students from Southwest China completed the SES scale, the normative beliefs about aggression questionnaire, the relational aggression behavior questionnaire, and the Chinese version of the Buss and Perry aggression questionnaire. Three main findings were obtained. First, SES showed a significant positive correlation with relational aggression (β = 0.22, *p* < 0.001) and a negative correlation with verbal aggression (β = −0.10, *p* < 0.001), but not with physical aggression (β = −0.02, *p* = 0.44). Second, normative beliefs about aggression mediated the relationships between SES and two types of aggression (physical and relational); anger/hostility mediated the relationships between SES and all types of aggression (verbal, physical, and relational). Finally, among boys, only the direct effect of SES on relational aggression and the mediating effect of normative beliefs about aggression were significant; however, among girls, the result was the same as that of the total sample. The findings provide support for social learning theory and the general aggression model, highlight the important role of SES in adolescent development, and clarify the distinct patterns underlying physical, verbal, and relational aggression. This study provides empirical evidence that may assist parents and that teachers can use to effectively intervene in adolescent aggressive behavior.

## 1. Introduction

Aggressive behavior is a complex social behavior that commonly occurs in different countries and among various groups of people. It also exists at almost every stage of human life, from birth to death (Tremblay, 2000). It can be categorized as direct aggression and indirect aggression (Lansford et al., 2012). The former is divided into physical aggression and verbal aggression. Physical aggression refers to aggressive behavior in which the attacker uses various parts of the body as tools to carry out a series of physical activities against others (Ji et al., 2020); verbal aggression refers to the aggressive behavior of hurting others through verbal language, such as insults and name-calling (Warburton & Anderson, 2015). Indirect aggression, also known as relational aggression, refers to an individual’s behavior of hurting others through evasive means, such as spreading rumors or speaking ill of people behind their backs (Coyne et al., 2006).

Adolescence is a critical period during which aggressive behavior becomes more frequent (Gómez-Ortiz et al., 2016; Tremblay, 2000). Many researchers have explored the influencing factors of individual aggressive behavior in adolescence from several perspectives, including family (Arim et al., 2011; Z. Li et al., 2022; P. Yang et al., 2022), community (Bradshaw et al., 2009; S. Lin et al., 2020), school (Mancini et al., 2006; Prati et al., 2018), peers (Y. Li & Guo, 2016; H. Liu et al., 2021; Y. Sun & Sun, 2021), and drug intake (Jesus Gazquez et al., 2016; Kivimaki et al., 2014; Kristjansson et al., 2021; White et al., 2013). This study focuses on the influence of SES on aggressive behavior in middle school students.

SES is a core variable affecting child development (H. Zhou, 2013) and reflects social stratification in modern societies (Zhu & Zhang, 2013). Previous studies have shown that SES is related to adolescents’ mental health (Reiss, 2013; Sulemana et al., 2016), physical health (Matthews & Gallo, 2011; Nusslock & Miller, 2016), and antisocial behavior (Aslund et al., 2013; Piotrowska et al., 2015). According to social learning theory, individuals learn behaviors and norms by observing others and the consequences of their actions (Bandura, 1989). Thus, differences in adolescents’ SES mean that there are differences in the educational levels and behavior that may shape behavioral development through variations in parental education, behavioral models, living environments, and social interactions.

In China, family plays a central role in socialization, and family is regarded as the basic unit of society. Therefore, exploring the relationship between SES and aggressive behavior among Chinese middle school students can provide important evidence for educators and parents seeking to reduce adolescent aggression.

Furthermore, the general aggression model proposes both long-term and short-term processes underlying aggressive behavior (Allen et al., 2018; Anderson & Bushman, 2002). Long-term exposure to violent environments may shape aggressive beliefs and affect, eventually forming aggressive personality traits. In the short term, environmental variables influence cognition, emotion, and physiological arousal, which, in turn, guide behavioral responses. According to the general aggression model, SES may influence aggressive behavior through cognitive and emotional mechanisms. Therefore, the present study examines both the direct relationship between SES and three types of aggression and the indirect effects through aggressive cognition and aggressive affect.

### 1.1. Socioeconomic Status and Aggressive Behavior

SES refers to the social standing of individuals or groups based on their access to social resources (Zhang et al., 2007). It is typically divided into subjective SES, reflecting individuals’ perceived social status (Davis, 1956; Singh-Manoux et al., 2003), and objective SES, commonly measured by family income, parental education, and parental occupation (Bradley & Corwyn, 2002; Matthews & Gallo, 2011). This study focuses on objective SES, which reflects differences in access to external resources.

Many studies have shown that SES is associated with aggressive behavior in adolescents (Henninger & Luze, 2013; Holtz et al., 2015; Park et al., 2005; Shameem & Hamid, 2014). Adolescents with lower SES tend to exhibit more frequent aggressive behavior than those with higher SES (Bonica et al., 2003; Shameem & Hamid, 2014; Qi & Kaiser, 2003). Family investment theory states that families with a lower SES may provide fewer developmental resources and opportunities, such as educational support (Matthews & Gallo, 2011; Conger et al., 2010), which can contribute to developmental difficulties in children (Brooks-Gunn & Duncan, 1997). Evidence also showed that family income and parental education are associated with children’s internalizing and externalizing behavioral problems (Evans, 2016; Votruba-Drzal, 2006; McLaughlin et al., 2011).

In China, research on social class psychology indicates that SES influences adolescents’ subjective well-being (Xing, 2011), life satisfaction (Zhang et al., 2007), psychological suzhi (Dong & Zhang, 2015), mental health (Cheng et al., 2019), and academic performance (Cheng et al., 2018). However, relatively few studies have empirically examined the relationship between SES and adolescent aggressive behavior.

Existing Chinese studies show inconsistent findings. For example, M. Lin (2018) found no significant predictive effect of SES on aggression among 658 high school students, possibly because aggression was measured using a total score that included cognitive and emotional components. Tao (2021) also reported no significant association between SES and several types of aggression among 577 students, which the author attributed to limited variability among participants. B. Yang (2021) surveyed 8992 Grade 8 students but used only four items to measure aggression, which may have limited the reliability of the results. Other studies examined SES indicators separately. For instance, parental education level was negatively associated with students’ aggression scores (Wang et al., 2021b), while some studies found no significant differences across educational levels (K. Xu, 2015). Similarly, parental occupation and family income showed mixed associations with different forms of aggression (Jiang, 2013). Although findings vary, these studies suggest that parental income, education, and occupation are all related to adolescent aggressive behavior, and it may be inappropriate to examine these factors in isolation.

### 1.2. Aggressive Cognition and Aggressive Affect

The general aggression model emphasizes the important roles that an individual’s cognition and emotions play in the relationship between environmental variables and behavioral outcomes. As a key family environment variable, SES may influence adolescents’ cognitive (Bradley & Corwyn, 2002; Dietze & Knowles, 2016) and emotional development (Gallo et al., 2005; Wu et al., 2015). Individuals with low SES often experience greater life stress and may develop higher vigilance and hostile cognition (Ouwehand et al., 2009; Gallo & Matthews, 2003; E. Chen et al., 2006). These cognitive patterns can increase negative emotions (Najman et al., 2010; Pascoe et al., 2015; Yoshikawa et al., 2012) and contribute to generating problematic behaviors (G.-z. Liu et al., 2020; Wu et al., 2015). In contrast, individuals with higher SES tend to report more positive emotions and more adaptive cognitive characteristics (Wu et al., 2015; B. Chen & Zhao, 2017; Burhan et al., 2017).

Normative beliefs about aggression represent an individual’s evaluation of the acceptability of aggressive behavior (Huesmann & Guerra, 1997). The more acceptable an individual feels aggressive behavior is, the more likely they are to carry out such behavior. Previous studies have shown that such beliefs predict aggressive behavior and may reinforce aggression over time (Duan et al., 2014; X. Sun et al., 2019; Krahé & Busching, 2014). Family environment also influences these beliefs, with supportive environments reducing adolescents’ aggressive behavior (Lv et al., 2019). Thus, normative beliefs about aggression may mediate the relationship between SES and aggressive behavior.

Anger is another important antecedent to aggressive behavior (Mackie et al., 2000). Numerous studies have revealed an important link between aggressive affect and aggressive behavior (Adachi & Willoughby, 2016; P. Li et al., 2022; Ying & Dai, 2008). For example, Sukhodolsky and Ruchkin (2004) found that higher anger levels were associated with more frequent physical aggression among juvenile offenders and middle school students. Similarly, relative deprivation theory suggests that disadvantaged individuals may experience anger and resentment after social comparison, which may lead to aggressive behavior (Smith et al., 2012).

### 1.3. Gender Differences

Gender differences in aggressive behavior have been reported by many researchers, but some of the findings are inconsistent. The findings indicated that males are consistently shown to be significantly more physically and verbally aggressive than females (Chung et al., 2019; Salimi et al., 2019; Wang et al., 2021b; Yeh et al., 2010), while some studies report no significant differences (Xin & Zheng, 2019). Research on relational aggression is also mixed. Some studies suggest that females display more relational aggression (L. Chen, 2012; Niu et al., 2016), whereas cross-national evidence indicates no consistent gender differences (Lansford et al., 2012). A longitudinal study finding further suggests that gender differences may vary across developmental stages (Lu et al., 2018). This implies that gender differences in individuals exhibiting aggressive behavior are conditioned.

Gender may also moderate the relationship between SES and behavioral problems. Zahn-Waxler et al. (2006) proposed that the types and mechanisms of behavioral problems shown by men and women may differ due to the influence of congenital and acquired factors. Supporting this view, a second-order meta-analysis including 474 studies and 327,617 participants found that gender moderated the association between SES and externalizing behavior problems (Korous et al., 2018). Additionally, gender differences in such behavioral problems may be moderated by race or ethnicity (Mclaughlin et al., 2007).

### 1.4. The Present Study

Previous studies have the following shortcomings.

Firstly, previous studies did not explore and clarify the entire relationship between SES and aggression in adolescents in a Chinese context. Meanwhile, they have not provided enough empirical evidence to determine whether the effects of SES on the three types of aggressive behavior in adolescents are consistent, especially given the differences between direct aggression and indirect aggression.

Secondly, previous studies did not demonstrate the intermediary role of normative beliefs about aggression between SES and aggressive behavior, nor did they demonstrate whether the relationship between normative beliefs about aggression and the three types of aggressive behavior is consistent. This deficiency also exists in the study of aggressive emotions.

Furthermore, we also wanted to test the differences in male and female groups to clarify the moderating effect of gender on the relationship between SES and aggressive behavior.

Therefore, this study proposes the following research questions:

Q1: Does SES have consistent effects on physical, verbal, and relational aggression in adolescents?
Q2: Do normative beliefs about aggression play a mediating role between SES and aggressive behavior? If so, are these roles similar across aggressive behavior types?
Q3: Does aggressive affect play a mediating role between SES and aggressive behavior? If so, are these roles similar across aggressive behavior types?
Q4: Does gender influence the association between SES and the three types of aggressive behaviors?

This study uses social learning theory and the general aggression model as the theoretical bases to explore the relationship between SES and three types of aggressive behavior (physical aggression, verbal aggression, and relational aggression) among adolescents in the context of Chinese culture. This study also investigates the roles of aggression cognition (i.e., normative beliefs about aggression) and aggressive affect (i.e., anger/hostility) as mediating variables in the relationship between SES and aggressive behavior by building a structural equation model. We also tested differences in male and female groups to clarify the moderating effect of gender on the relationship between SES and aggressive behavior. The results will help researchers clarify the link between SES and aggressive behaviors and the important role of SES in adolescent development.

## 2. Methods

### 2.1. Participants

In total, 1891 adolescent students (male students = 51.2%; n = 969) from Southwest China were selected. Among them, 873 were in the seventh grade, 814 were in the eighth grade, and 204 were in the ninth grade. Participants’ ages ranged from 12 to 15 years (*M* = 13.06 years, *SD* = 0.84). Most participants (59.5%; 1126 students) reported that they were not an only child, while 40.5% (765 students) reported that they were an only child. Meanwhile, 15.0% (284 students) reported that they lived in rural areas, and 85.0% (1607 students) reported that they lived in urban areas. Moreover, 29.0% (548 students) were left-behind children^1^, and 71.0% (1343 students) were non-left-behind children. It is worth noting that to effectively reduce participants’ defensive responses and mitigate social desirability bias, we used bold font on the first page to inform participants that their responses were anonymous, that the data would be used only for scientific research, and that strict confidentiality would be maintained.

### 2.2. Measures

#### 2.2.1. Socioeconomic Status

Following existing research (G.-z. Liu et al., 2020; F. Xu et al., 2009), the parental education level, parental occupation, and family monthly income were used as sub-dimensions of SES. Parental education level was divided into primary school and below, junior middle school, high middle school or technical secondary school, college for professional training, and undergraduate and postgraduate, which were assigned the numbers 1 to 6, respectively. Family monthly income includes 2000 yuan and below, 2000–3000 yuan, 3000–4000 yuan, 4000–5000 yuan, 5000–6000 yuan, 6000–7000 yuan, 7000–8000 yuan, 8000–9000 yuan, 9000–10,000 yuan, and more than 10,000 yuan (assigned numbers from 1 to 10, respectively). Parental occupations included 13 options: farmers, workers, doctors, teachers or researchers, officers or civil servants, lawyers, accountants, military, engineers, enterprise managers, individual/private entrepreneurs, individual household, or unemployed persons and volunteers. According to the degree of professionalism of the occupation, the secondary coding of the occupation is divided as follows: peasant or jobless, blue collar, and professional or semiprofessional (Fuligni & Zhang, 2004), which were assigned the numbers 1, 2, and 3, respectively. Following relevant research (F. Xu et al., 2009), parental educational level, parental occupation, and family monthly income were first standardized in the statistical analysis; then, we calculated their average as an SES. In the present study, the Cronbach’s alpha was 0.81.

#### 2.2.2. Normative Beliefs About Aggression

The scale used to measure the normative beliefs of participants about aggression was compiled by Huesmann and Guerra (1997) and revised by Shao and Wang (2017) for its localization in China. The scale consists of 20 items, divided into two dimensions: approval of retaliation and general approval of aggression. Subjects were asked to rate the correctness of the behavior described by each item (such as “In general, you think of beating someone else”) using a four-point scale (1 = very wrong, 4 = very correct). The higher the score, the higher the individual’s level of normative beliefs about aggression (i.e., the more agreeable aggressive behavior is). In the present study, the Cronbach’s alpha was 0.85.

#### 2.2.3. Aggressive Affect

Following existing studies (Adachi & Willoughby, 2016; Swing & Anderson, 2014), the dimensions of anger (7 items, such as “Sometimes I lose my temper for no reason”) and hostility (8 items, such as “I am wary of strangers who treat me too kindly”) in the Buss and Perry aggression questionnaire (Buss & Perry, 1992) were combined into the dimension of aggressive affect. The scale was scored on the five-point Likert scale (1 = strongly disagree, and 5 = strongly agree). In this study, the final score was averaged. The higher the score, the higher the individual’s level of aggressive affect (anger and hostility). In the present study, the Cronbach’s alpha was 0.77.

#### 2.2.4. Aggressive Behavior

Verbal aggression/physical aggression were measured using the verbal/physical aggression dimension of the Buss and Perry aggression questionnaire (Buss & Perry, 1992). They contain 5 items (such as “My friends say that I love to argue with people”) and 9 items (such as “I would do that if I had to use force to protect my rights”), respectively. The scale was scored on a five-point Likert scale (1 = strongly disagree, and 5 = strongly agree). In this study, the final score was averaged. The higher the score, the higher the individual’s level of verbal or physical aggression. In the present study, the Cronbach’s alpha was 0.54 and 0.70 for verbal aggression and physical aggression, respectively.

Relational aggression was compiled based on Liang (2005) and comprised 9 items, such as “After quarreling with someone, speaking ill of that person in front of others…” and “When someone makes me angry, I ignore him/her for a short time…”. All items were reverse-scored, using the five-point Likert scoring (1 = this item is very consistent with me, 5 = this item is not consistent with me at all). In this study, the final score was averaged. The higher the score, the stronger the relational aggressive behavior of the individual. In the present study, the Cronbach’s alpha was 0.93.

### 2.3. Data Analytic Strategy

First, with missing values (1.6% to 9.5% of the sample) in this study, we analyzed the data using the full information maximum likelihood estimator^2^ (FIML) by Mplus 8.3. Second, we used Mplus 8.3 to analyze mediating effects and multi-group analysis.

In addition, we prevented common method bias from interfering with the research results by adopting Harman’s univariate test (Podsakoff et al., 2003) to measure the degree of common method variability. The results showed that there were 14 eigenvalues greater than 1 in all factors, and the variance explained by the first factor was 22.95%. The test results conform to the judgment conditions proposed by Ashford and Tsui (1991), which means that common method bias did not affect the statistical analysis and results in this study.

## 3. Results

### 3.1. Descriptive Statistics and Correlations

Table 1 summarizes the study variables for the whole sample. The skewness and kurtosis values for the study variables met the criteria for normality. SES was significantly positively correlated with aggressive cognition and relational aggression and significantly negatively correlated with aggressive affect and verbal aggression. Aggressive cognition was significantly positively correlated with physical aggression and relational aggression and significantly negatively correlated with verbal aggression. Aggressive affect was significantly positively correlated with verbal aggression and physical aggression and significantly negatively correlated with relational aggression. Relational aggression was significantly negatively correlated with verbal aggression and physical aggression. Verbal aggression was significantly positively correlated with physical aggression.

### 3.2. Difference Test of Variables in Demography

Previous studies have shown that adolescent aggression can be influenced by factors such as grade level, place of residence, only-child status, and left-behind status. For example, Wang et al. (2021a) found significant differences in aggression among primary and secondary school students based on gender, only-child status, family residence, parental education, parenting style, and grade level. Similarly, Pi et al. (2025) reported that left-behind children exhibit higher levels of aggressive behavior than non-left-behind children, indicating that left-behind status may be an important factor related to adolescent aggression. Therefore, this study included these demographic variables as controls in the data analysis. Additionally, to clarify the control variables in the mediation model, we also tested the differences in the demographic information of each study variable. The results showed that the SES was significantly different depending on the grade (*F*_(2, 1888)_ = 80.60, *p* < 0.001), family residence (*t*_(642)_ = −25.40, *p* < 0.001), and whether a child was an only child (*t*_(1887)_ = −14.23, *p* < 0.001). Normative beliefs about aggression were significantly different based on the grade (*F*_(2, 1890)_ = 57.45, *p* < 0.001), gender (*t*_(1853)_ = 4.61, *p* < 0.001), family residence (*t*_(348)_ = −26.92, *p* < 0.001), and whether a child was an only child (*t*_(1884)_ = −10.52, *p* < 0.001). Aggressive affect was significantly different depending on the grade (*F*_(2, 1890)_ = 5.40, *p* < 0.01), gender (*t*_(1858)_ = −3.65, *p* < 0.001), family residence (*t*_(1889)_ = 2.56, *p* = 0.01), and whether a child was an only child (*t*_(1889)_ = 3.57, *p* < 0.001) or a left-behind child (*t*_(1889)_ = 4.18, *p* < 0.001). Verbal aggression was significantly different depending on grade (*F*_(2, 1890)_ = 4.09, *p* < 0.001), family residence (*t*_(1889)_ = 6.22, *p* < 0.001), and whether a child was an only child (*t*_(1889)_ = 2.39, *p* = 0.02) or a left-behind child (*t*_(1889)_ = 3.10, *p* < 0.01). Physical aggression was significantly different depending on the grade (*F*_(2, 1890)_ = 3.48, *p* = 0.03), gender (*t*_(1889)_ = 4.34, *p* < 0.001), and whether a child was a left-behind child (*t*_(1889)_ = 2.12, *p* = 0.03). Relational aggression was significantly different depending on the grade (*F*_(2, 1890)_ = 81.86, *p* < 0.001), gender (*t*_(1858)_ = 2.66, *p* < 0.01), family residence (*t*_(468)_ = −36.45, *p* < 0.001), and whether a child was an only child (*t*_(1836)_ = −9.84, *p* < 0.001).

Based on the results presented above, we considered the grade, gender, family residence, and whether a child was an only child or a left-behind child as control variables in the following structural equation model.

### 3.3. Structural Equation Modeling for the Total Sample

First, we tested the direct effect between the independent variable and the dependent variable (Wen & Ye, 2014). The results indicated a good model fit (χ^2^/*df* = 1.20, *RMSEA* = 0.01, *CFI* = 0.99, *TLI* = 0.99, and *SRMR* = 0.01). After controlling for the grade, gender, only-child status, family residence, and left-behind child status, SES had a significant correlation with verbal aggression (β = −0.10, *p* < 0.001) and relational aggression (β = 0.22, *p* < 0.001), but had no significant correlation with physical aggression (β = −0.02, *p* = 0.44).

**Figure 1:**
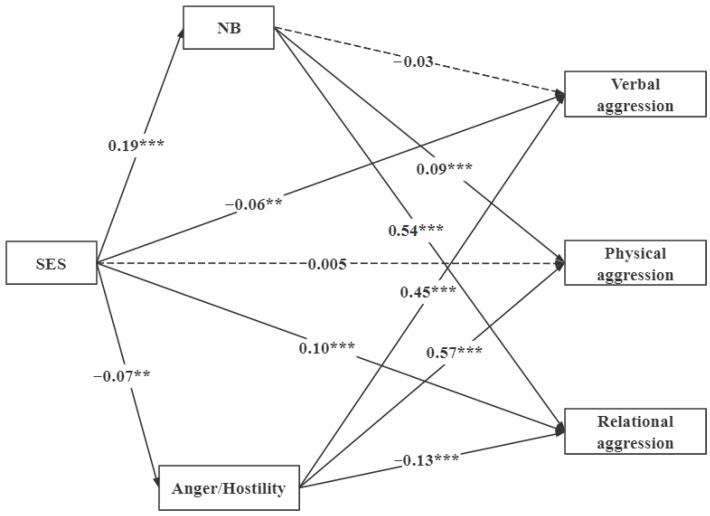
Structural equation model for the full sample. Note: Standardized coefficients presented. NB: Normative beliefs about aggression. ** *p* < 0.01, *** *p* < 0.001.

Then, we added aggressive cognition and aggressive affect as mediating variables and found that the new model had a good fit (χ^2^/*df* = 3.57, *RMSEA* = 0.04, *CFI* = 0.99, *TLI* = 0.97, *SRMR* = 0.01). Figure 1 shows the results after controlling for the grade, gender, only child, family residence, and being a left-behind child.

Three noteworthy findings emerged. First, SES had a significant positive correlation with aggressive cognition (β = 0.19, *p* < 0.001) and relational aggression (β = 0.10, *p* < 0.001) and a negative correlation with aggressive affect (β = −0.07, *p* = 0.007) and verbal aggression (β = −0.06, *p* = 0.009). As before, it had no significant correlation with physical aggression (β = 0.005, *p* = 0.79). Second, aggressive cognition had a significant positive correlation with physical aggression (β = 0.09, *p* < 0.001) and relational aggression (β = 0.54, *p* < 0.001) but had no significant correlation with verbal aggression (β = −0.03, *p* = 0.204). Third, aggressive affect had a significant positive correlation with predicted verbal aggression (β = 0.45, *p* < 0.001) and physical aggression (β = 0.57, *p* < 0.001) and a negative correlation with relational aggression (β = −0.13, *p* < 0.001).

Using the bias-corrected bootstrap method, we generated 5000 samples. Two notable results were found (see Table 2). First, the 95% confidence interval of the mediating effect of normative beliefs about aggression between SES and verbal aggression included 0, while the 95% confidence interval of the mediation effect test of SES and physical aggression and relational aggression did not include 0. This outcome indicates that normative beliefs about aggression mediate SES’s relationships with physical aggression and relational aggression. Second, the 95% confidence interval of aggressive affect in the three mediation tests did not include 0, which indicates that aggressive affect mediates SES’s relationships with verbal aggression, physical aggression, and relational aggression.

### 3.4. Structural Equation Modeling for Boys and Girls

**Figure 2:**
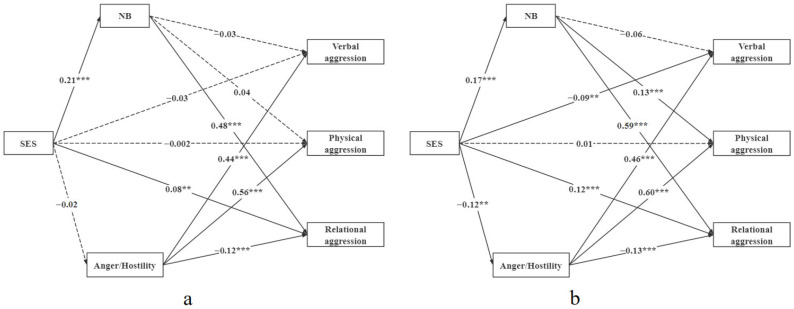
Structural equation model for different genders. (**a**) Structural equation model for the male sample. (**b**) Structural equation model for the female sample. Note: Standardized coefficients presented. NB: Normative beliefs about aggression. ** *p* < 0.01, *** *p* < 0.001.

As shown in Figure 2, the male and female samples were tested separately in accordance with the method of the above mediation effect model test. According to the model fitting index, both the male and female samples can accept two models (see Table 3), which can be grouped by gender so that cross-group comparisons can be made between models. Therefore, based on the mediation model, gender differences were included in the model.

A multi-group comparison was used to test the gender differences in the structural equation model. The results are shown in Table 3. Chi-square differences (Δχ^2^ = 23.62, *p* < 0.05) were found between the free estimation model (M1) and the restricted path model (M2). The results showed significant differences between boys and girls in the mediation models.

Subsequently, the path coefficients of the two models were limited so that they would be equal, and the results showed that the regression coefficient between normative beliefs about aggression and relational aggression in boys (β = 0.49, *p* < 0.001) was significantly lower than that in girls (β = 0.59, *p* < 0.001).

## 4. Discussion

### 4.1. The Main Findings

This study is one of the first to systematically examine the direct and indirect links between SES and aggressive behavior in adolescents, as well as the potential role of gender in these links within the Chinese cultural context. It can expand the existing framework of SES-aggressive behavior research, which is primarily based on Western samples. The findings answer the four questions posed in the introduction. Specifically, SES directly predicts verbal aggression and relational aggression, but not physical aggression, among middle school students. SES also indirectly influenced physical aggression and relational aggression through aggressive cognition, whereas aggressive affect mediated all three pathways. Furthermore, the relationship between SES and aggressive behavior differed between males and females. Interestingly, relational aggression was negatively correlated with verbal and physical aggression among adolescents. This finding is inconsistent with previous studies (Gaertner et al., 2010; Ettekal & Ladd, 2017). One possible explanation is that adolescents tend to choose either direct or indirect aggression when engaging in aggressive behavior, as this study measured the frequency of aggressive behaviors.

### 4.2. SES and Verbal Aggression

SES showed a significant negative correlation with verbal aggression among middle school students. Furthermore, it could only indirectly affect verbal aggression through the mediating effect of aggressive affect, while the mediating effect of aggressive cognition was not significant. That is, adolescents with low SES have stronger anger and hostility than those with high SES, which makes them tend to use verbal aggression to carry out violent acts. This result aligns with the relative deprivation theory proposed by Smith et al. (2012). Because parents with low SES are not able to invest enough in their children’s education (Conger et al., 2010), adolescents with low SES do not have strong emotional regulation skills, hindering their positive development (Matthews & Gallo, 2011). Therefore, low SES groups are more passive (He, 2010) than high SES groups, and they vent their negative emotions as verbal aggression.

### 4.3. SES and Relational Aggression

We also found that SES directly and indirectly influenced relational aggression through aggressive cognition and aggressive affect. In contrast to verbal aggression, adolescents with higher SES showed higher levels of relational aggression, which is consistent with previous studies (Bonica et al., 2003; McNeilly-Choque et al., 1996). Some researchers believed that the more complex a person’s social relationships were, the more likely they were to commit relational aggression (Xia et al., 2017). Compared with individuals with lower SES, those with higher SES often value their own reputation and interpersonal evaluations more strongly (Hart & Edelstein, 1992; Weininger & Lareau, 2009). Thus, they tend to try to harm others indirectly. According to social learning theory, adolescents with a high SES learn relational aggressive behavior patterns from their parents or the surrounding environment, which they internalize as their own behavioral norms, thus accepting this indirect aggressive behavior pattern. A high social class background is conducive to individual independence (Carey & Markus, 2017). So, once such normative beliefs about aggression are established, they are difficult to change (Huesmann & Guerra, 1997). In similar situations, adolescents with high SES had a relational aggression script activated to exhibit their approved relational aggression.

Meanwhile, the results showed that SES was negatively associated with aggressive affect, and lower aggressive affect negatively predicted relational aggression in adolescents. This finding differs from previous research on anger and relational aggression (Ersan, 2020). Families with a high SES can provide teens with more material or emotional support, or the parents of such families can exhibit emotion management strategies that teens can learn to use to reduce their anger and maintain their positive emotions (C. Zhou & Guo, 2013). However, low anger/hostility can predict high relational aggression among adolescents, which is inconsistent with Ersan (2020). A possible reason for this discrepancy is that the subjects of Ersan’s research were preschool children, whereas the subjects of the present study were middle school students. The concept of developmental psychology holds that middle school students have a strong self-centered mental performance, they attach great importance to the evaluations of others, and they believe that they are unique and always in the spotlight of others. At the same time, individuals with a high SES pay more attention than those with a low SES to other people’s impressions of themselves (Hart & Edelstein, 1992; Weininger & Lareau, 2009). Thus, to maintain a good impression of themselves in the minds of others, individuals with a high SES feel they cannot show direct, observable aggressive behavior. They may even actively control their emotional expressions and not show their anger and hostility to others in front of others. Therefore, they have to choose the covert form of relational aggression to achieve their purpose. Of note, adolescents need support from their peers when spreading rumors and excluding others (Card et al., 2008), and those with a high SES have such resources.

### 4.4. SES and Physical Aggression

SES showed no significant correlation with the physical aggression of middle school students. Further, the predictive effects of the mediating effects of aggressive cognition and aggressive affect were the opposite, which showed a masking effect. This finding is inconsistent with existing research results (Bellair et al., 2019; Piotrowska et al., 2015). There are three possible reasons for this outcome. First, Chinese social norms discourage direct physical conflict (Zhao & Zhou, 2010), leading adolescents across SES groups to display similarly low levels of physical aggression. Second, relational aggression and verbal aggression are more covert and may allow perpetrators to avoid punishment from teachers or parents (J.-z. Chen et al., 2013; Vitaro et al., 2006). Third, individuals with higher SES may maintain stable normative beliefs while also developing stronger emotional regulation skills, leading to the opposite mediating effects of cognition and affect.

### 4.5. Gender Differences in the Relationship Between SES and Aggressive Behavior

Gender differences were examined in the relationships between SES and the three types of aggression. Specifically, the female model is similar to the overall model, with differences observed only in the coefficients of each path. Meanwhile, the male model shows that the SES significantly positively predicts relational aggression and indirectly affects the relational aggression of individuals through the mediator of normative beliefs about aggression. Moreover, compared with the female model, the coefficient between normative beliefs about aggression and relational aggressive behavior in the male model was lower. This is similar to many studies showing that women show more relational aggression than men.

The results of the above gender differences showed clear socio-cultural roles in two ways. First, social requirements and norms for men differ from those for women. In the thousand-year-old historical background of Chinese culture, men are required to be elegant and easy-going rather than violent. In Confucian culture, a good male should be a gentleman who is modest and prudent, who can strictly command himself, and who has a noble character. Second, society does not pay enough attention to relational aggression and does not have a deep understanding of the harm caused by relational aggression (X. Liu & Liu, 2020). Parents’ beliefs that relational aggression should be punished less severely than physical aggression (E. Goldstein & Boxer, 2013) affects the formation of children’s self-perceptions, creating an illusion that relational aggression is reasonable.

### 4.6. Theoretical and Practical Implications

In summary, based on social learning theory and the general aggression model, this study explored the relationships of SES with adolescents’ verbal aggression, physical aggression, and relational aggression in the context of Chinese culture. Some differences and similarities were detected between the results of this study and those of international research. The results showed that middle school students with a high SES were more likely to use relational aggression, while middle school students with a low SES were more likely to use verbal aggression. Our results also suggest that gender moderates the association between SES and the three types of aggressive behavior that we investigated.

Based on our results, we issue three warnings. First, the family environment has a predictive effect on adolescent aggressive behavior (Lei & Wen, 2021; Lv et al., 2019), but its influence on different types of aggressive behavior is inconsistent. Thus, we should not generalize the aggressive behavior in the future, but should carry out targeted research. Second, middle school students’ social and cultural backgrounds significantly influence their acquisition and performance of aggressive behavior (Zhong et al., 2014), and individuals of different genders reflect this influence differently. Thus, parents and teachers should pay more attention to relational aggression exhibited by middle school students, especially boys, as opposed to focusing solely on their direct aggressive behavior. Third, schools should set up personal files of students to help teachers understand and master each student’s family background, so as to give targeted help measures.

### 4.7. Limitations and Future Research

This study also has several limitations. In terms of measurement, this study also has several limitations. In terms of measurement, adolescents reported their parents’ income, education, and occupation, which may introduce bias in assessing objective SES. Parental occupations were also categorized into three broad groups, which may obscure socioeconomic heterogeneity. Additionally, standardized SES indices such as the Hollingshead Index or the Family Affluence Scale were not used, limiting comparability with other studies. Future research should employ standardized SES measures and incorporate parent-reported data.

In terms of data results, the Cronbach’s alpha for verbal aggression was relatively low, a problem also reported in previous studies (B. Chen & Zhao, 2017; B. Chen et al., 2018). This may reflect limitations of the questionnaire itself, which has not undergone major revisions for many years despite social changes. Updating the scale may improve its measurement accuracy. In terms of variable selection, although objective SES is an important factor influencing adolescent aggression, subjective SES may also influence emotional and behavioral outcomes (Sweeting & Hunt, 2014). Compared with objective resource disparities, perceived disadvantage may more directly activate cognitive and emotional processes such as anger, hostility, and aggressive cognition. Future research could include both objective and subjective SES in the same model and incorporate indicators of peer status or social network position to better test the proposed mechanisms. Finally, because this study used cross-sectional data, causal relationships between SES and adolescent aggression cannot be determined. Future research should employ longitudinal designs to clarify these causal relationships.

## 5. Conclusions

The results confirmed that the SES of middle school students affects their expressions of physical aggression, verbal aggression, and relational aggression. Furthermore, we found that aggression cognition and aggression affect played a mediating role, respectively, and that gender moderates the relationships among the above variables. In the context of Chinese culture, adolescents with a high SES tend to use relational aggressive behavior, while adolescents with a low SES tend to use verbal aggressive behavior; physical aggression is not a preference in either group. Moreover, for boys, SES predicted only their relational aggression. The findings provide an empirical basis for the rationality of social learning theory and the general aggression model. At the same time, the results suggest that parents and teachers should pay attention to the impact of SES on the development of adolescents and treat different types of aggressive behavior differently.

## Figures and Tables

**Table 1:** Pearson correlation analysis and descriptive statistics among variables.

	1	2	3	4	5	6
1. SES	1					
2. NB	0.16 **	1				
3. AA	−0.05 ***	−0.01	1			
4. VA	−0.08 ***	−0.04 ***	0.21 ***	1		
5. PA	−0.01	0.03 ***	0.24 ***	0.20 ***	1	
6. RA	0.39 ***	0.72 **	−0.12 ***	−0.19 ***	−0.09 ***	1
*M*	0.01	2.27	2.60	2.68	2.30	3.60
*SD*	0.57	0.25	0.37	0.53	0.47	1.46
Skewness	0.09	−1.13	0.30	0.18	0.47	−0.75
Kurtosis	−1.04	1.06	−0.13	−0.11	−0.01	−0.83

Note. SES: Socioeconomic status; NB: Normative beliefs about aggression; AA: Aggressive affect; VA: Verbal aggression; PA: Physical aggression; RA: Relational aggression. ** *p* < 0.01, *** *p* < 0.001.

**Table 2:** Tests of bootstrapped standardized intermediary effects.

		β	*SE*	*t*	Bootstrap 95% *CI*		Relative Mediating Effect
					Lower	Upper	
VA							
	Direct effect	−0.06	0.02	−2.62	−0.11	−0.02	60%
	Indirect effect 1	−0.01	0.01	−1.11	−0.01	0.004	10%
	Indirect effect 2	−0.03	0.01	−2.66	−0.06	−0.01	30%
PA							
	Direct effect	0.01	0.02	0.27	−0.04	0.05	-
	Indirect effect 1	0.02	0.005	3.60	0.01	0.03	-
	Indirect effect 2	−0.04	0.02	−2.69	−0.07	−0.01	-
RA							
	Direct effect	0.10	0.02	5.62	0.07	0.14	47.62%
	Indirect effect 1	0.10	0.01	8.03	0.08	0.13	47.62%
	Indirect effect 2	0.01	0.004	2.52	0.003	0.02	4.76%

Note. VA: Verbal aggression; PA: Physical aggression; RA: Relational aggression. Indirect effect 1: The mediating role of normative beliefs about aggression; indirect effect 2: The mediating role of aggressive affect.

**Table 3:** Model fit.

	Fit Indices							
	χ^2^	*df*	*CFA*	*TLI*	*RMSEA*	*SRMR*	∆χ^2^ (*M*2 − *M*1)	∆*df* (*M*2 − *M*1)
Male	15.85	6	0.99	0.96	0.04	0.02		
Female	8.22	6	0.99	0.99	0.02	0.01		
M1	24.07	12	0.99	0.98	0.03	0.01		
M2	47.69	23	0.99	0.98	0.03	0.02	23.62	11

## Data Availability

The datasets generated and/or analyzed during the current study are available from the corresponding author on reasonable request.

## Notes

1 Left-behind children refer to minors under the age of 18 who are left behind in rural areas where they are registered, unable to live with their parents.

2 This study also used the method of mean interpolation of missing values to process the data, and the results were basically consistent with the current presentation.
